# Waiting and Care in Pandemic Times Collection

**DOI:** 10.12688/wellcomeopenres.15971.1

**Published:** 2020-06-10

**Authors:** Lisa Baraitser, Laura Salisbury

**Affiliations:** 1 Department of Psychosocial Studies, Birkbeck University of London, London, WC1E 7HX, UK; 2 Department of English and Film, University of Exeter, Exeter, Devon, EX4 4QJ, UK

**Keywords:** COVID-19, UK Government, waiting, time, care, lockdown

## Abstract

This editorial introduces a collection of research articles and reflections on what it means to wait during the time of the COVID-19 pandemic. Written from conditions of lockdown, this collection gathers together the initial thoughts of a group of interdisciplinary scholars in the humanities and social sciences who have been working on questions of waiting and care through a project called *Waiting Times*.

In early March 2020, a meme began to circulate on social media that used a quotation from J.R.R. Tolkien’s *The Fellowship of the Ring* (1954), part of *The Lord of the Rings* trilogy voted the UK’s favourite novel in a BBC poll in 2003. As they wait in anticipation of the tasks that lie ahead, the hobbit Frodo says to the wizard Gandalf: ‘“I wish it need not have happened in my time”’. Gandalf replies: ‘“So do I, and so do all who live to see such times. But that is not for them to decide. All we have to decide is what to do with the time that is given us”’ ( Tolkien, 1974, p. 78). As is well known, Tolkien’s conservative and romanticised version of a modest English courage found in hobbits, alongside his vision of a world torn apart by technologies of war, was profoundly influenced by his experience in the trenches of World War I and then living through a second global conflict. The meme is clearly meant both to convey regret that anyone should live to see times shot through with such profound losses and to inspire courage in the face of what lies ahead, while comfortingly positioning its readers as up to the task.

The question of ‘what to do with the time that is given us’ in the current moment of the coronavirus pandemic is certainly pressing, both for those thrust into conditions of impotence and anxious waiting in which there seems to be little to be done, and for those expected to act under conditions of urgency and emergency. But the idea that people understand that the current crisis has somehow released time – a time that might be used but that might also be dangerously misused – is worth attending to. What would it mean to look at COVID-19 not just as a public health and political crisis that requires action, but also a crisis of time – a moment where questions of temporality and its relation to care have come urgently to the fore?

This collection of papers represents the initial thoughts of a group of interdisciplinary scholars in the humanities and social sciences who have been working on questions of waiting and care through a project called *Waiting Times*, funded by the Wellcome Trust. We begin from the understanding that waiting is one of healthcare’s core experiences. It is there in the time it takes to access services; through the days, weeks, months or years needed for diagnoses; in the time that treatment takes; and in the elongated time-frames of recovery, relapse, remission and dying. Our aim in this project is to open up what it means to wait in and for healthcare by examining lived experiences, representations and histories of delayed and impeded time. Contextualising healthcare practices within broader social organisations of time allows us to grasp the meanings, potentialities and difficulties of waiting in current times. The aim of the research is to move beyond thinking focused on the urgent need to reduce unnecessary waiting times in the UK’s National Health Service (NHS), towards a more comprehensive understanding of the relation between waiting, care and changing experiences of time.

We are writing from the experience of lockdown in the UK in March and April 2020, where whole populations have been instructed that waiting at home in order to ‘flatten the curve’ of the outbreak is a form of care – for selves, for others and for the institution of the NHS. And we have been asking ourselves how a longer history and broader perspective on delayed and impeded temporalities might help to make sense both of the potentialities and dangers of these current waiting times. Lisa Baraitser and Laura Salisbury address this question by thinking through the terms used by the UK Government to describe their response to the pandemic: containment, delay and mitigation. Through a psychosocial reading of each term, they outline how the difficulties inherent within waiting might be used to help understand the relationship between time and care and the necessity of paying attention to the ever-present possibility of violence and failures of care *within* acts of care.

Martin Moore historicises the appeal to ‘save the NHS’ in the current pandemic in the context of longer-term anxieties about the service’s capacity to survive increasing demand and public and policy discourses that have framed it as being ‘at risk’. The paper therefore works to understand the current discourses about waiting in order to protect the NHS as part of a longer history in which time has been experienced and understood as both a threat to the service and its capacity to care, and a way of managing or caring for an institution with a potent place in the national imaginary.

Stephanie Davies was just beginning ethnographic research in a GP surgery in Hackney, London, when its first case of COVID-19 was reported and treated. Her paper explores what it might mean for a service already experiencing itself as in a chronic crisis of funding and capacity to come into contact with another crisis of time and of care.

Jocelyn Catty writes from her experience as a child and adolescent psychotherapist working in the NHS to ask what happens to the offer of time, care and rhythmic continuity that sits at the core of psychoanalytic psychotherapeutic practice, under conditions of emergency. She notes how adolescents’ experience of anxious pressure and uncertainty, particularly in the face of a future that seems unable to unfold in ways that could be productively used, is now mirrored in experiences of a broader population held in the waiting time of lockdown.

Jordan Osserman and Aimée Lê use a quite different tradition of psychoanalytic thinking to work through what failures of authority might mean in the context of COVID-19. Teasing out the relationships between time and power, and writing from the position of those in precarious employment, Osserman and Lê suggest that in these current waiting times – in the suspension of ‘business as usual’ – there is potential for radical action and for a reconfiguration of the socio-political sphere that would place care at its core.

Michael J. Flexer also argues that the time of COVID-19 represents a distinct, but currently unexamined, temporal moment. Using semiotic methods, he traces how the mechanical actions of the virus, through becoming social, create what he names as ‘a new viral time’ – a time that reveals that we have already arrived in a new historical epoch. This epoch, he argues, holds revolutionary potential. While the current socio-economic order attempts to reimpose temporal certainty and fixity through hastily conceived political actions, it is the possibility for profound re-imaginings of our productive and social relations that should concern us most.

Finally, Martin O’Brien’s essay considers the relationship between the experience of life shortening chronic illness and the current COVID-19 crisis, using his own experience of living with cystic fibrosis to interrogate the temporal experience within the global pandemic. Having now lived beyond his own life-expectancy, he draws on his own concept of ‘zombie time’ to understand the presence of death as a way of life. O’Brien’s art practices form the basis for his analysis of living through a pandemic that mimics his own sick and coughing body. O’Brien argues that we are currently occupying a widespread zombie time, which frames other people as carriers of death. It is only through finding new ways of being together that we can survive.

## Data availability

No data is associated with this article.
